# Expanding boundaries – a cell biologist's guide to expansion microscopy

**DOI:** 10.1242/jcs.260765

**Published:** 2024-04-17

**Authors:** Nadja Hümpfer, Ria Thielhorn, Helge Ewers

**Affiliations:** Department of Biology, Chemistry and Pharmacy, Institut für Chemie und Biochemie, Freie Universität Berlin, 14195 Berlin, Germany

**Keywords:** Expansion microscopy, Hydrogel, Super-resolution microscopy

## Abstract

Expansion microscopy (ExM) is a revolutionary novel approach to increase resolution in light microscopy. In contrast to super-resolution microscopy methods that rely on sophisticated technological advances, including novel instrumentation, ExM instead is entirely based on sample preparation. In ExM, labeled target molecules in fixed cells are anchored in a hydrogel, which is then physically enlarged by osmotic swelling. The isotropic swelling of the hydrogel pulls the labels apart from one another, and their relative organization can thus be resolved using conventional microscopes even if it was below the diffraction limit of light beforehand. As ExM can additionally benefit from the technical resolution enhancements achieved by super-resolution microscopy, it can reach into the nanometer range of resolution with an astoundingly low degree of error induced by distortion during the physical expansion process. Because the underlying chemistry is well understood and the technique is based on a relatively simple procedure, ExM is easily reproducible in non-expert laboratories and has quickly been adopted to address an ever-expanding spectrum of problems across the life sciences. In this Review, we provide an overview of this rapidly expanding new field, summarize the most important insights gained so far and attempt to offer an outlook on future developments.

## Teaser

**Summary:** Expansion microscopy is a sample preparation technique that allows increased resolution in many settings by physically enlarging specimens and can be easily adopted in cell biology laboratories.

## Introduction

Expansion microscopy (ExM) was invented in the laboratory of Ed Boyden in 2015 (Chen et al., 2015). It is a technique whereby a dense, swellable polyelectrolyte hydrogel is created within a fixed specimen and crosslinked chemically to its components. When the hydrogel is then expanded, it physically pulls the sample components apart in all spatial directions, creating a much larger facsimile of the specimen. As a result, when this facsimile is examined using microscopy, the apparent resolution with respect to the original sample is increased to the same degree as the hydrogel has expanded (approximately fourfold in the original work). Molecules that previously were too close to each other to be resolved are now separated far enough to be detected independently, according to the respective expansion factor. Although this approach might sound too simple to work, it does so strikingly well and has quickly been reproduced in many laboratories.

Accordingly, adoption of this technique has led to an explosive growth in the development of alternative and improved approaches, and ExM has since been applied successfully to a wide range of biological systems and microscopy approaches. At a fast pace, beyond the development of a simplified protocol for imaging of proteinaceous structures in cells (Chozinski et al., 2016; Tillberg et al., 2016) and formulations that allow for up to 20-fold expansion (Chang et al., 2017), ExM has been adapted for use with tissue samples for pathological investigation of material from patients (Zhao et al., 2017), as well as nucleic acids (Chen et al., 2016). This revolutionary technique has been very quickly brought into mainstream cell biology, and ExM data have been published in a wide range of fields including plant biology, cell biology and synaptic connectomics (Arya et al., 2022; Cirillo et al., 2020; Comer et al., 2020; Deshpande et al., 2017; Hafner et al., 2019; Kao and Nodine, 2019; Lee et al., 2019; Liffner et al., 2023; Mosca et al., 2017; Sathe et al., 2018; Suofu et al., 2017; Wang et al., 2020). Importantly, these exemplary studies are not from laboratories that are focused on ExM or the development of microscopy techniques, demonstrating the ease of transferability of ExM. At the same time, groups at the forefront of microscopy assay development have quickly adapted ExM to a vast variety of existing techniques for higher-resolution imaging towards the nanometer scale (Gao et al., 2018; Halpern et al., 2017; Louvel et al., 2023; Shaib et al., 2023 preprint; Xu et al., 2019; Zwettler et al., 2020), as well as higher expansion factors (Chang et al., 2017; Damstra et al., 2022; Klimas et al., 2023; Louvel et al., 2023; M'Saad and Bewersdorf, 2020; Truckenbrodt et al., 2018), aimed at imaging of entire vertebrate embryos (Sim et al., 2022 preprint). Furthermore, gel embedding and expansion have proven to be compatible with, and increase spatial resolution in, spatial transcriptomics (Alon et al., 2021) and mass spectrometry imaging (Bai et al., 2023; Ku et al., 2016). However, as is to be expected, this new technique has also brought unique challenges related to hydrogel chemistry, the process of expansion itself and the need for homogenization of the sample. Careful controls for isotropic expansion in all spatial dimensions are essential for reliable results. Here, we summarize the progress made so far in improving the ExM technique, applying it to different systems, ensuring sufficient labeling and accurate data analysis, and transferring it to novel applications. Furthermore, we provide a brief guide to well-documented information in the literature that is helpful for understanding the technical foundation of the technique; extensive reviews of the chemistry underlying ExM approaches have been published elsewhere (Truckenbrodt, 2023; Wen et al., 2023a) and detailed protocols are available (see Table S1). Our aim is to provide an accessible overview of the field for the interested cell biologist.

## A brief overview of the principle of ExM

**Fig. 1.**
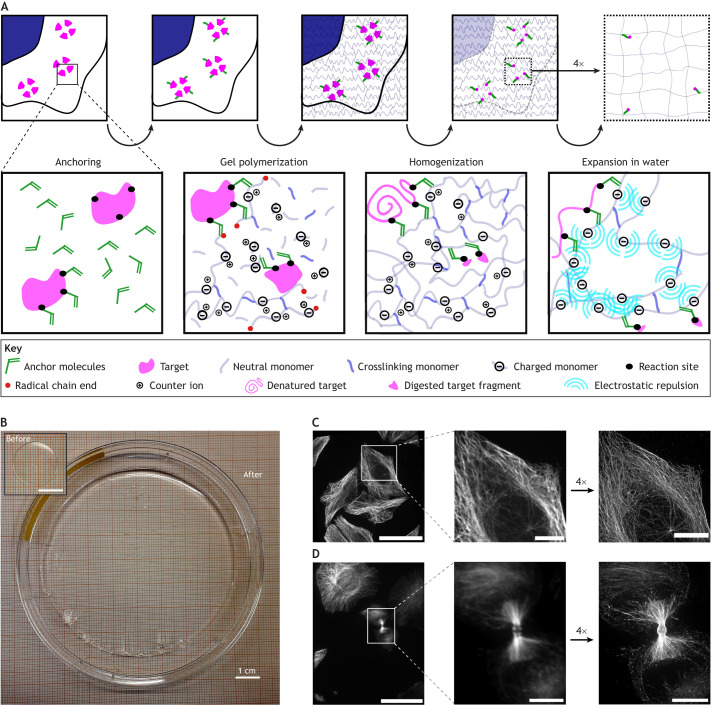
**The principle of ExM.** (A) The top row shows a schematic illustration of the expansion process in a cell, with the nucleus shown in blue. Three protein complexes are located in the cytoplasm. The broken magenta rings symbolize structures too small to be resolved by light microscopy. The constituting molecules are chemically coupled to a reagent (‘anchors’, green) that can be crosslinked to a polymerizing hydrogel (anchoring). Upon initiation of gel polymerization by free radicals, the anchors become incorporated into the hydrogel (gray) by formation of covalent bonds (gel polymerization). The sample is then degraded or denatured, leaving the gel behind with the crosslinked positions of the target molecules as a facsimile of their original positions (homogenization). When the hydrogel is then swollen through the addition of water, the target molecules are pulled apart, leading to a higher apparent resolution of the sample, which can be exploited in standard microscopy methods (expansion in water). Staining can be applied prior to gel formation or after the expansion. The bottom row illustrates, from left to right, the molecular processes underlying anchoring, gel polymerization, homogenization and expansion. Anchoring molecules (green), such as AcX, react with functional groups in the structure of interest (such as primary amines in proteins, represented by black dots in the magenta structure). For gel polymerization, a solution of different monomers is soaked into the sample. Traditionally, this contains mono-reactive uncharged monomers (gray), such as acrylamide; bi-reactive monomers for crosslinking (blue), such as bisacrylamide; and mono-reactive charged monomers (symbolized by ‘−’), such as sodium acrylate, with their counter ions (‘+’), such as Na^+^. Polymerization is initiated by radicals (red dots) and leads to the formation of a three-dimensional gel with the anchors incorporated. Homogenization using heat and detergent denatures the protein (top left), whereas homogenization using proteinase digestion leads to fragmentation of the protein, with only parts remaining coupled to the hydrogel (bottom right). The swelling of the gel in water is attributed to the negative charges in the polymer; when the cation counterions are washed away, electrostatic repulsion (cyan arcs) forces the chains apart. Shielding of charges by water molecules (not depicted) leads to swelling of the hydrogel. (B) Image of an expanded hydrogel with the original size of the gel shown in the inset (top left), taken by Nadja Hümpfer. (C) The image on the left shows a fluorescence micrograph of unexpanded HeLa M cells with microtubules stained by immunofluorescence, alongside an enlarged image of the region indicated by a white box. The image on the right shows the resolution improvement for the same region after fourfold expansion. Scale bars: left, 50 μm; middle, 10 μm; right, 40 μm. (D) The image on the left shows a fluorescence micrograph of an unexpanded HeLa M cell in cytokinesis, with microtubules stained by immunofluorescence, alongside an enlarged image of the region indicated by a white box. The image on the right shows that post-expansion immunofluorescence staining allows visualization of previously inaccessible epitopes in the midbody, owing to decrowding of the midbody as a result of expansion. Scale bars: left, 50 µm; middle, 10 µm; right, 40 µm. Images in C and D taken by N.H. and Thilo Brill (Freie Universität Berlin).

Although a large number of different approaches have been published, the sample preparation process of ExM can be roughly divided into the following common steps, as illustrated in Fig. 1A. (1) Anchoring: a fixed sample is incubated with a crosslinking agent that links the target molecule (either a biomolecule that will be labeled post expansion or a previously introduced label) to the polymerizing gel. (2) Polymerization: the sample is immersed in a solution of monomers consisting of some that carry charges and others that create crosslinks between growing polymer strands; this solution forms a hydrogel through radical polymerization. The target molecules that bear a crosslinker from the first step are covalently linked to this gel during polymerization. This process is called grafting in polymer chemistry. (3) Homogenization: the sample is homogenized though enzymatic digestion or a combination of heat and detergent treatment; this removes the multitude of non-covalent interactions that organize the integrity of the cell and allows for isotropic expansion of the gel. (4) Expansion: immersion of the gel in deionized water leads to repulsion of the charges incorporated into the hydrogel, resulting in a maximal extension of the polymer chains the gel is formed from; the result is a hydrogel that is swollen in all three dimensions and when imaged yields a higher resolution (Fig. 1B,C). A number of detailed protocols have been published for the different approaches (Asano et al., 2018; Bucur et al., 2020; Truckenbrodt et al., 2019), so we will only briefly describe the key steps below.

### Anchoring

ExM involves significant chemical and mechanical alteration of the sample as its components become incorporated into a newly formed gel. ExM is thus incompatible with live cells, and the first step in the process is cell fixation. Following that, the sample is prepared for crosslinking with the polymer by the addition of reactive anchors that can form covalent bonds with the hydrogel. Proteins are commonly crosslinked to the polymer by a bivalent crosslinker, whereby a polymerizable monomer domain, such as acrylate (Tillberg et al., 2016) or methacrylate (Chozinski et al., 2016), is introduced into the polymerizing gel, while a succinimidyl ester group allows linkage to free amines on proteins. Alternatively, glutaraldehyde (Chozinski et al., 2016) and tetrafluorophenyl (TFP) ester (Wen et al., 2020) have proven to be potent crosslinkers. Fixation of the sample using low a concentration of formaldehyde in the presence of acrylamide has been shown to increase the accessibility of antigens for immunostaining and structural preservation of multiprotein complexes (Gambarotto et al., 2019; Ku et al., 2016; Sarkar et al., 2022). Recently, glycidyl methacrylate has been proposed for the effective anchoring of protein and RNA simultaneously (Cui et al., 2023). Furthermore, to directly crosslink (graft) only target molecules to the sample and label them at the same time, multifunctional dyes have been developed (Thielhorn et al., 2023; Wen et al., 2020, 2023b).

### Polymerization and grafting

After covalently attaching the anchors for incorporation into the polymerizing gel, a mixture of hydrogel-forming monomers is soaked into the sample (Chen et al., 2015). This mixture usually contains acrylamide as the backbone of the gel, a charge bearing monomer such as sodium acrylate to ensure charge–charge repulsion-driven expansion of the gel and a crosslinking monomer such as bisacrylamide for gel stability. Key factors, such as the expansion factor and the mechanical stability of the gel, depend on the ratio between the charge-bearing monomer, the neutral backbone monomer and crosslinker density (see Table S2 and references therein). Gel formation depends on the formation of radicals, which then interact with each other through diffusion. It is therefore crucial that the reagents are homogenously mixed before the reaction is started. The reaction is started by the addition of so-called radical starters, as are also used to prepare acrylamide hydrogels for electrophoresis. This leads to growth of many polymer chains from monomers in the solution by collision. Here, the reaction of some growing chains with anchoring molecules on the target structure to bring about the so-called surface grafting of target molecules and/or their labels into the gel is essential, as otherwise the target molecules cannot be visualized. Most common mixtures used in ExM contain very high concentrations of monomers, with approximately one monomer per nanometer in all dimensions. This means that a growing polymer chain is much more likely to collide and react with a freely diffusing monomer than with an anchoring molecule on a target structure (Thielhorn et al., 2023). Consequently, the incorporation of monomers on the surface of target molecules into the polymerizing gel (surface grafting) is relatively unlikely, as polymerization occurs through diffusion-mediated collision. To effectively link all material of the sample into the polymerizing gel, a high concentration of anchoring agent must be used in sample preparation, or several anchors must be attached to a target molecule (Thielhorn et al., 2023; Wen et al., 2023b).

As the chemistry of hydrogel formation is well-established, variations of the basic formulations have been quickly developed that allow, for instance, an increased expansion factor (see below; Damstra et al., 2022; Truckenbrodt et al., 2018) or changes in other properties of the hydrogel (see Table S1). Such variations include the use of alternative hydrogel-forming monomers and bivalent crosslinkers, or changes in the balance between the key gel components (i.e. the monomer, charge-bearing monomer and crosslinker).

Clearly, a homogenous progression of the polymerization reaction is essential for an evenly formed gel and thus isotropic expansion later on. This is especially challenging for high-volume samples such as tissues. To trigger polymerization evenly, 4-hydroxy-2,2,6,6-tetramethylpiperidin-1-oxyl (often abbreviated as 4-HT or 4-hydroxy-TEMPO; Chen et al., 2015) can be added as a polymerization inhibitor. In this way, the sample can be penetrated evenly by the gelation solution in a longer pre-incubation of the sample in monomer solution before polymerization. Alternatively, polymerization speed can be reduced by the lowering of temperature (Song et al., 2018).

### Sample homogenization

After the gel is formed, the sample is homogenized to remove all intermolecular interactions that are not mediated by the gel in order to allow for isotropic expansion (Chen et al., 2015). Here, broad-specificity proteases, such as proteinase K, can be used to digest proteins, leaving only small peptides that, by chance, are anchored into the gel (unanchored peptides are otherwise washed away). Alternatively, homogenization can also be performed using heat and detergents, which allows for epitopes to be retained (even if in a denatured form) for post-expansion labeling. It is essential that the sample is completely homogenized, as incomplete homogenization can lead to tearing of the gel, especially at higher expansion factors (Truckenbrodt et al., 2019). These homogenization methods are targeted at proteins, whereas nucleic acids are denatured by heat but not cut by proteases, and lipids are only partially fixed under most conditions (Tanaka et al., 2010) and removed by detergent. As a result, membranes as entities cannot survive the process of expansion. How membranes and nucleic acids are stained will be discussed in detail further below.

### Expansion

After the gel is formed in the sample and the sample is homogenized, the gel is placed in deionized water. The ions in the buffer that previously shielded charges from each other are washed away, and the negative charges of the acrylate polymer repel one another. As a result, the previously tightly intertwined and wrinkled hydrogel network becomes maximally stretched to allow for the highest possible distance between charges. The resulting increase in volume is stabilized by incorporation of water molecules between the polymer chains, resulting in a gel that expands in all spatial dimensions by a factor of four to five (Chen et al., 2015).

At this stage, the sample is a hydrogel in which either denatured polypeptide chains or their significantly digested fragments are covalently attached to the hydrogel matrix to form a larger facsimile of the specimen (see Table S2 for an overview of the parameters that control the expansion process). Target molecules in the sample may be stained by fluorescent probes before or after expansion and can now be imaged. Both the significant processing of the sample and the nature of the now much larger sample lead to specific challenges posed by this new technology, as discussed below.

## Challenges associated with ExM and how they are being addressed

**Fig. 2.**
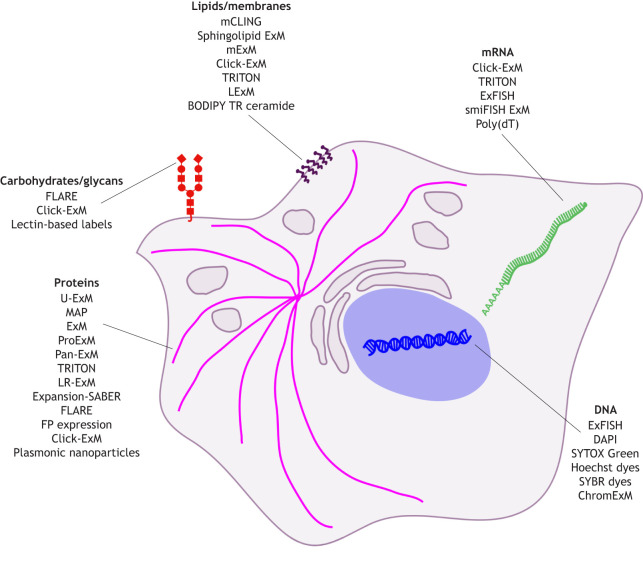
**Overview of labeling methods and dyes targeted at specific cellular components that have been used with ExM.** Schematic illustration of labeling approaches that have been established for ExM of the main cellular components: carbohydrates (red), lipids (purple), DNA (blue), RNA (green) and proteins (magenta). Some labeling approaches are compatible with multiple cellular components. A detailed explanation of all the labeling methods, their pitfalls and references can be found in Table S3. ChromExM, chromatin ExM; Expansion-SABER, ExM with signal amplification by exchange reaction; FP, fluorescent protein; LExM, lipid ExM; LR-ExM, label-retention ExM; MAP, magnified analysis of the proteome; mCLING, membrane-binding fluorophore–cysteine–lysine–palmitoyl group; mExM, membrane ExM; poly(dT), poly-deoxythymidine; ProExM, protein retention ExM; U-ExM, ultrastructure ExM.

ExM is an elaborate sample preparation technique that creates higher resolution through physical enlargement of the sample rather than a microscopy technique. ExM was originally developed to gain access to obstructed epitopes in dense multiprotein complexes by pulling the constituent molecules apart, and it can indeed improve the staining of such structures (see Fig. 1D for an example). The chemistry of hydrogel formation, the homogenization of the sample and the expansion to a much higher volume all lead to challenges that are not necessarily unique to ExM but must be addressed for successful staining. These challenges are discussed below. Expansion is, in principle, compatible with traditional staining methods such as immunofluorescence. Although proteinase K treatment will result in degradation of all proteins, protocols exist for the retention of the structure and function of at least some fluorescent proteins (Tillberg et al., 2016), including those capable of photoswitching for use in super-resolution photoactivated localization microscopy (PALM) (Betzig et al., 2006). However, since the expansion of the sample by, for instance, a factor of four in one dimension leads to a 64-fold increase in volume, ExM necessarily results in a lower signal intensity, as the fluorophore labels are distributed over a larger volume after expansion. As a result, several approaches for highly intense labeling have been developed, with the simplest being the addition of multiple labels with the same dye (Gao et al., 2018). In addition, post-staining signal amplification can be performed, either through biotin–streptavidin labeling (Kim et al., 2019; Karagiannis et al., 2019 preprint; Sun et al., 2021) or via template-multiplication-based DNA labeling, where DNA oligonucleotides delivered via antibodies are labeled by the addition of overlapping, reverse-complementary oligomers bearing dyes that polymerize into bright beacons (Saka et al., 2019). Besides the unavoidable dilution of labels, sample homogenization can lead to the destruction of proteinaceous fluorophores and other labeling agents, such as antibodies (Chen et al., 2015). Similarly, some dyes can undergo irreversible bleaching during the chemically aggressive radical-mediated polymerization step (Chen et al., 2015; Chozinski et al., 2016; Min et al., 2020; Tillberg et al., 2016), or they may be lost through incomplete grafting into the gel. Consistent with this, the introduction of fluorophores post expansion results in a stronger signal (Chozinski et al., 2016; Gambarotto et al., 2019; Ku et al., 2016; Shi et al., 2021); however, in this case, the primary antibodies used for immunofluorescence should be reactive towards the denatured protein, such as those used for detection in western blotting, as homogenization leads to protein denaturation. Therefore, a successful ExM experiment relies on a well-thought-out staining protocol, and a variety of optimized staining approaches have been published (Fig. 2; Table S3).

Since most ExM protocols focus on crosslinking of proteins, other types of molecules – such as nucleic acids, lipids and carbohydrates – must be considered specifically for labeling. A specific problem with lipid labeling, for example, is that lipids are not incorporated into the gel, as most do not contain primary amines; therefore, the staining of membranes in ExM mainly relies on click chemistry and metabolic labeling (M'Saad and Bewersdorf, 2020; Sun et al., 2021; White et al., 2022), or the introduction of amphiphilic probes with an ‘anchorable’ amine group (Damstra et al., 2022; Götz et al., 2020; Karagiannis et al., 2019 preprint). A ceramide-coupled dye has been reported to also yield successful staining (Liffner and Absalon, 2021). The staining of RNA has been achieved in a technique called ExFISH, which combines the commercial nucleic acid labeling reagent Label IT with 6-[(acryloyl)amino]hexanoic acid succinimidyl ester (Acryloyl-X SE, also known as AcX) for anchoring into the gel and fluorescence labeling via regular fluorescence *in situ* hybridization (FISH) probes (Chen et al., 2016). For brighter labeling, many RNA FISH probes that bind to small stretches of the target and contain a so-called ‘handle sequence’ can be combined with many dye-coupled oligomers against that handle sequence in a technique called single-molecule inexpensive FISH (smiFISH; Tsanov et al., 2016). These techniques are, however, aimed at specific target sequences; for global DNA labeling, common DNA-intercalating dyes, such as DAPI, Hoechst, SYBR and SYTOX dyes, have been found to work well in ExM (Chozinski et al., 2016; Min et al., 2020; M'Saad and Bewersdorf, 2020). More complex labeling procedures such as Click-ExM and trivalent anchoring (TRITON) (Sun et al., 2021; Wen et al., 2020) are also compatible with nucleic acid staining.

Pan-ExM uses an entirely different labeling approach; here, non-specific probes such as succinimidyl ester (NHS)-coupled fluorophores stain the entire protein or lipid content of the cell or tissue, resulting in the generation of contrast evoked by all cellular proteins and giving rise to images that resemble those obtained using electron microscopy (Liffner and Absalon, 2021; M'Saad and Bewersdorf, 2020; M'Saad et al., 2022 preprint). Both DNA-intercalating dyes and NHS-coupled dyes are combined with chemical labeling of carbohydrates using periodate–hydrazide labeling in a technique named fluorescent labeling of abundant reactive entities (FLARE), which thus allows global staining of DNA, protein and carbohydrates (Mao et al., 2020). Alternatives for carbohydrate labeling include lectins, which bind to specific sugars and, as proteins, are linked into the gel, as well as the use of click chemistry via hydrazide-coupled dyes (Klimas et al., 2023; Ku et al., 2016).

The heterogeneity of biological samples with regard to their molecular composition not only complicates labeling but also poses challenges for the expansion process itself. In biological specimens, proteins, nucleic acids, carbohydrates, proteoglycans and lipids are often distributed unevenly, especially in complex samples such as tissues or entire organisms. The nucleus, for example, contains most of the DNA in the cell, whereas cell wall structures or the outer shell of insects are composed of thick layers of difficult-to-digest material. This inhomogeneous distribution of the components of a specimen gives rise to non-uniform mechanical properties and chemical environments. As a result, incomplete homogenization can lead to areas that are resistant to expansion, resulting in tearing. These problems have been overcome in preparations of major model organisms, many pathogenic microorganisms, organoids and even patient tissues with the development of specific protocols suited to the particular specimen (see Table S4 for a list of published protocols). For example, nuclei and spindle-aligned chromosomes appear to expand well (Chozinski et al., 2016), but chromosomal substructures can appear distorted by ExM (Kubalová et al., 2020). This might be due to local distortions caused by the high charge density of DNA or the fact that DNA molecules remain intact following homogenization for ExM and thus cannot stretch in all dimensions. These problems may be overcome by nuclease treatment before expansion of the crosslinked sample, as has been employed to investigate the structural organization of mitotic chromosomes, whose structure is based on a complex set of DNA-associated proteins (Xu et al., 2019). In another application, chitinase has been used to digest the body wall of *Drosophila* larvae (Jiang et al., 2018), and here, polymerization is temporarily blocked to allow for complete soaking of monomers into the tissues before gel formation.

As hydrogel formation is well understood, its weaknesses are also known. In particular, gels generated by free-radical crosslinking polymerization, such as the acrylamide-based gels most often used in ExM, are prone to network heterogeneities that result from the stochastic, diffusion-based nature of radical-induced polymerization (Lorenzo and Seiffert, 2015). Polymerizing chains can form loops or unconnected free ends, and regions rich in crosslinks can expand to a lesser extent than those with a lower crosslinker density. As a result, gel expansion is not homogeneous (Aoki et al., 2000; Cohen et al., 1992). Furthermore, the local protein content within the sample can influence the expansion factor, leading to a difference in expansion factor both between protein structures within a cell and between the cell and the surrounding medium (Büttner et al., 2021; Martínez et al., 2020; Pesce et al., 2019).

To benchmark an ExM protocol, the achieved expansion factor of the sample can be determined by imaging the same region of interest of the sample both before and after expansion and comparing the results. This can be done either on the level of specific individual features or the entire image by, for instance, comparing nuclear size and area before and after expansion (Faulkner et al., 2022), or using photobleaching of defined areas before expansion to assess any distortions introduced during expansion (Vanheusden et al., 2020). Another option is the creation or incorporation of regular patterns, such as fluorescent substrates (Damstra et al., 2023; Nakamoto et al., 2022) or photocleavage of labels in a specific pattern (Pownall et al., 2023). If the shape of such patterns changes after expansion, distortions in the gel must have been introduced (Box 1).

**Box 1. Limitations and pitfalls of ExM**

Although ExM is, in general, a straightforward method to establish in the laboratory, finding optimal conditions for homogeneous and isotropic expansion of the sample and successful staining can be challenging. In addition, the experimental procedure is time consuming, as it can take days to complete. The quality of the reagents, as well as anchoring and homogenization conditions, play an important role in preservation of intact structures. Conditions that are not optimal for the target specimen can lead to tears in the gel as it expands – as illustrated in the left-hand image of the box figure, which shows an immunostained culture of hippocampal neurons with arrowheads indicating a tear in the gel [scale bar, 50 µm; image is reproduced from Truckenbrodt et al. (2019), with permission from SNCSC, and is not published under the terms of the CC-BY-4.0 license of this article; for permission to reuse, please see individual reference] – or more treacherously, to anisotropic expansion, resulting in more difficult to detect artifacts. Indeed, it has been reported that different cellular organelles can exhibit differing expansion factors in the same cell (Büttner et al., 2021). It is thus of greatest importance that proper controls are included in the experimental design. The middle and right-hand images in the box figure show fluorescence micrographs of the same cell nucleus taken before and after expansion [reproduced from Vanheusden et al. (2020) under the terms of a CC-BY 4.0 license]. Here, a square field in the center of the nucleus was photobleached before expansion. Although at first glance the nucleus seems to have expanded isotropically, in the image taken after expansion the shape of the photobleached area exhibits a clear distortion at the bottom-left corner (marked by arrows; Vanheusden et al., 2020). Such comparisons between images taken before and after expansion are essential to control for isotropic expansion, especially during first optimization of the procedure. An alternative would be the inclusion of a size standard, such as DNA origami nanorulers (Lee et al., 2021; Scheible and Tinnefeld, 2018 preprint), or the seeding of cells on a reference pattern (Damstra et al., 2023). Ultimately, local differences in the chemical composition of a sample can lead to variation in how effectively the sample is grafted into the gel, thus resulting in uneven stress during expansion and leading to tears in the gel or uneven expansion. Protocols must be optimized to the sample at hand, particularly when imaging entire organisms, which exhibit strong local differences in the mechanical and chemical properties of their tissues (see Table S4). Another essential factor is the quality of the reagents used, which must be maintained to ensure a quantitative and even performance of the gelation reaction. Acrylamide, for example, must be stored in cold and dry conditions as it reacts to form dimers, and this reaction approximately doubles with every 5°C increase in temperature (Wampler, 1988). For a selection of quantitative studies assessing the factors that control gel properties see Table S2.
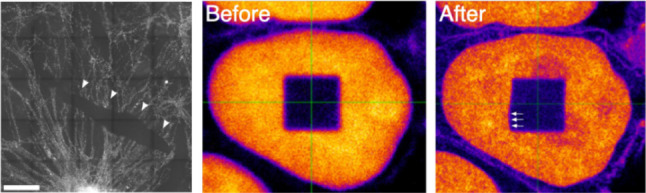

The physical enlargement of a sample creates unique challenges for light microscopy that are aggravated for higher expansion factors. Gels of larger samples can reach centimeters in size and, due to their nature as hydrogels, are delicate. The handling of hydrogels can be simplified by using imaging chambers to avoid drift, dehydration and tearing of the gels (Seehra et al., 2023; Shaib et al., 2023 preprint). Large gels are of course also expanded in the *Z*-direction and thus require deeper optical penetration into the sample, necessitating water immersion for high-quality imaging (Jurriens et al., 2021) or an adjustment of the immersion conditions by refractive index matching (Gao et al., 2018). Furthermore, optical distortions can become problematic further away from the coverslip when imaging with oil immersion. To avoid this, the challenges presented by thicker samples can be overcome by mechanical sectioning with a vibratome. Using a gel with increased rigidity, this technique, termed expansion tomography (ExT), has been successfully employed for high-throughput light-sheet tomography of mouse brains (Chen et al., 2021). Of course, the sheer size of the expanded gel often does not allow imaging of the sample in a single field of view, and ‘stitching’ several fields of view into a single image is required; this further necessitates highly homogenous illumination and, ideally, automated imaging. As a result, light-sheet imaging is increasingly being used for ExM (Bürgers et al., 2019; Chen et al., 2020; Düring et al., 2019; Gao et al., 2019; Lillvis et al., 2022; Lu et al., 2023; Mascheroni et al., 2020).

## New opportunities offered by ExM

**Fig. 3.**
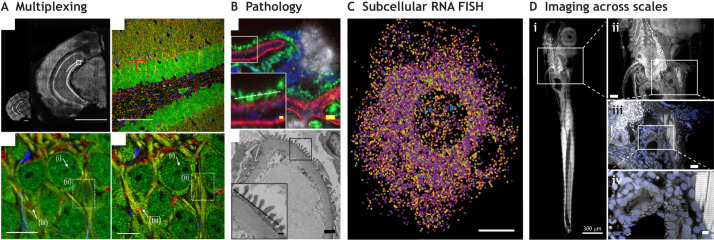
**Examples of ExM applications.** The original ExM principle has been applied to an extensive array of systems and techniques beyond imaging of cultured cells, as exemplified here. (A) Multiplexed ExM against many targets, shown here in a mouse hippocampal slice. Top left, overview images of the hippocampal slice, showing two slices at the same scale before (left) and after (right) expansion. White box indicates the region shown in the top-right image. Scale bars: 1 mm (left), 1 cm (right). Top right: Multiplexed labeling imaged before expansion. The astrocyte marker GFAP is in blue; NeuN, as a marker for postmitotic neurons, is in green; MAP2, as marker for dendrites, is in yellow; and MBP, as marker for myelin and/or oligodendrocytes, is in red. Red box indicates the region shown in the images beneath. Scale bar: 100 µm. Bottom row: the same region imaged before (left) and after (right) expansion. Weak expression of NeuN inside the nucleolus is revealed by expansion (arrow i), and dense dendrites (marked by MAP2, box ii) and the hollow structure of myelin (marked by MBP, arrow iii) are resolved. Scale bars: 10 µm (left), 30 µm (right). Images reprinted from Min et al. (2020), with permission from Elsevier. (B) An example of pathological investigation of material from humans using ExM. Minimal change disease (MCD) is usually diagnosed using electron microscopy. ExM of material from an individual without MCD (top) shows the same degree of detail as electron microscopy (bottom). In an individual with MCD, the clearly distinguishable thin foot processes would be flat. Detail of the foot processes revealed in ExM is sufficient to detect the disease. Stained in the ExM fluorescence micrograph are vimentin (blue), actinin (green), collagen IV (red) and DNA (DAPI, gray). Boxes indicate regions shown in insets, and dashed lines mark the base of the foot processes. Scale bars: 1 µm; insets, 200 nm. Reproduced from Zhao et al. (2017), with permission from SNCSC. (C) ExFISH signals of six different RNAs in a HeLa cell. Scale bar: 20 µm. Reproduced with permission from Chen et al. (2016), with permission from SNCSC. (D) Whole-body ExM of a zebrafish larva at 6 days post fertilization stained to detect total protein using Alexa Fluor 488–NHS (grayscale) and with nuclear staining (DAPI, blue). Subpanels showing images of increasing magnification (ii to iv) reveal that subcellular resolution is indeed achieved on the level of the entire larva. Scale bars: 300 µm (i), 50 µm (ii), 20 µm (iii) and 5 µm (iv). Reproduced from Sim et al. (2022 preprint), with permission of the copyright holder. Fig. 3 is not published under the terms of the CC-BY-4.0 license of this article. For permission to reuse, please see individual references.

The extremely fast adoption of ExM in a wide variety of imaging applications, with subjects ranging from molecular superstructures to entire organisms, clearly demonstrates the potential of this approach (see Fig. 3). Current super-resolution imaging techniques require significant expertise, as is the case for single-molecule localization microscopy (SMLM), or expensive dedicated equipment, as in the case of stimulated emission depletion (STED) microscopy; however, ExM avoids these issues while achieving a similar resolution. Both STED and SMLM are difficult to set up for multicolor imaging, a feature that is easily achieved when using ExM (Fig. 3A). The ease of combining ExM with existing light microscopy techniques allows for compatibility with open-source solutions that make high-resolution microscopy possible at a fraction of the cost of commercial confocal microscopy (Diederich et al., 2020; Hohlbein et al., 2022; Martens et al., 2019; Zhang et al., 2016, 2017). The imaging modality will be further simplified when higher expansion factors are combined with contrast methods such as silver staining instead of fluorescent dyes, which will allow imaging without the need for separate excitation and emission channels (M'Saad et al., 2022 preprint). Therefore, it can be expected that ExM methods combined with open-source microscopy approaches will greatly reduce the cost for many routine assays in diagnostic applications that rely on any form of imaging (Bucur et al., 2020; Zhao et al., 2017) (Fig. 3B), or high-throughput microscopy (Day et al., 2023 preprint), as ExM has been shown to be compatible with these applications.

One further important aspect is the applicability of ExM to complex samples that are inaccessible to high-resolution imaging by other means, such as cells on patterned nanosurfaces or even titanium implants that are inaccessible to some microscopy techniques (Nakamoto et al., 2022). In addition to uses in imaging, the expansion of tissue might also make it possible to perform spatially resolved proteomics with improved resolution. Here, the method used for tissue homogenization is especially important, as use of non-specific proteinase K would make identification of peptides after expansion impossible. Indeed, less aggressive homogenization, for example with the protease LysC (Drelich et al., 2021; Li et al., 2022b), allows identification of a large fraction of peptides. Similar to the use of tissue expansion in spatially resolved proteomics, ExM combined with *in situ* reverse transcription and sequencing has been shown to enable spatial transcriptomics with a resolution that is increased by the achieved expansion factor (Alon et al., 2021), and RNA FISH combined with ExM can localize many transcripts at nanoscale resolution (Chen et al., 2016) (Fig. 3C).

As discussed above, homogeneous gel formation is crucial for an even expansion of the sample. To that end, new monomer mixtures have been developed to ensure that an equally spaced polymer is formed upon gelation. For instance, the use of two different types of compatible tetrahedral monomers, each of which can only react with four molecules of the other type of monomer, creates a necessarily isotropic network (Gao et al., 2021), leading to lower distortion (Lee et al., 2021) and making the gel pore size controllable. Here, polymerization is based on click chemistry and therefore avoids undesired reactions that can result in the destruction of fluorescent dyes in radical-based polymerization reactions.

## Increasing resolution further

As discussed above, ExM increases resolution by physically expanding a sample. Of course, these expanded samples may then be imaged using super-resolution microscopy techniques to further increase resolution, and indeed, the major super-resolution techniques have been successfully combined with ExM, including structured illumination microscopy (SIM) (Cahoon et al., 2017; Halpern et al., 2017), STED (Gambarotto et al., 2019; Gao et al., 2018; Kim et al., 2019; Li et al., 2018; Pesce et al., 2019; Unnersjö-Jess et al., 2017) and, more recently, SMLM (Chang et al., 2023; Shi et al., 2021; Xu et al., 2019; Zwettler et al., 2020). These approaches face specific challenges resulting from the nature of expanded samples. For example, due to their size, expanded sample require very long imaging times for three-dimensional STED scanning, which at the desired resolution necessitates complete immobilization of the gel. Furthermore, most dye-switching-based SMLM approaches require buffers that are incompatible with the need for deionized water in the expansion protocol. The presence of ions in the imaging buffer results in gel shrinkage, which can be counteracted by re-embedding the expanded and stained gel in a neutral acrylamide gel that prevents shrinkage in the buffers used for dye-switching protocols.

**Fig. 4.**
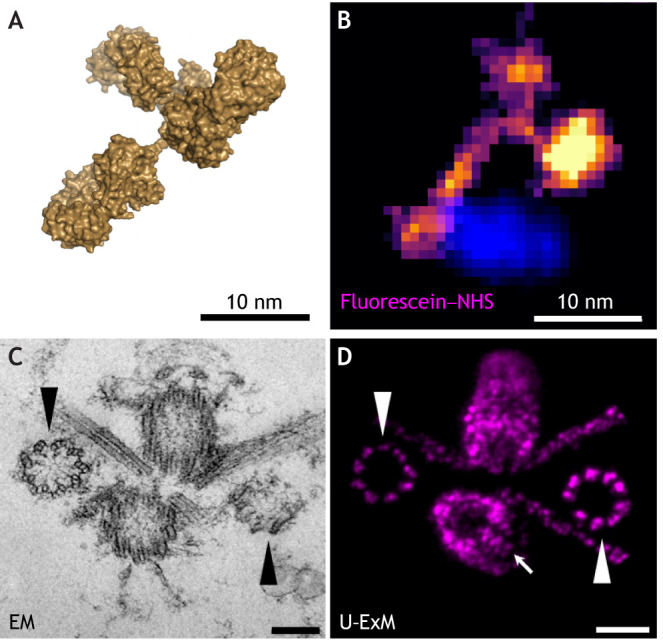
**Potential of ExM to image single proteins and multiprotein complexes.** (A) Surface rendering of an IgG molecule (PDB:1IGT). (B) One-nanometer expansion (ONE) microscopy image of a single IgG molecule shown at the same scale as in A. Reproduced from Shaib et al. (2023 preprint), with permission of the copyright holder. (C) Transmission electron microscopy (EM) image of a *Chlamydomonas* centriole pair. (D) Ultrastructure ExM (U-ExM) image of a centriole pair arranged similarly emphasizes the resolution that can be achieved. Arrowheads in C and D show the ninefold symmetry resolved in EM and U-ExM. Arrow in D indicates the mature centriole. Scale bars: 200 nm (C) and 800 nm (D). C and D reproduced from Gambarotto et al. (2019), with permission from SNCSC. Fig. 4 is not published under the terms of the CC-BY-4.0 license of this article. For permission to reuse, please see individual references.

Another means to improve resolution is by increasing the expansion factor (Truckenbrodt et al., 2018). Indeed, only two years after publication of the initial ExM protocol, it was shown that the expansion factor could be increased to ∼20-fold by applying two subsequent rounds of expansion to the same specimen (i.e. digestion of the first gel and fixing the initially expanded sample into a second hydrogel that is then expanded) (Chang et al., 2017). Less complex approaches have achieved expansion factors of ∼10-fold by modifying the polyacrylamide gel formulation (Damstra et al., 2022; Klimas et al., 2023; Li et al., 2022a; M'Saad and Bewersdorf, 2020; Truckenbrodt et al., 2018). Recently, cryo-fixation has been combined with ExM to circumvent the typical artifacts that are introduced by chemical fixation, such as shrinkage and breakdown of small membraneous and proteinaceous structures (Laporte et al., 2022). Recently, an increased expansion factor in combination with the use of software that facilitates fluctuation-based single-molecule detection, such as super-resolution optical fluctuation imaging (SOFI) (Dertinger et al., 2009) or super-resolution radial fluctuations (SRRF) (Gustafsson et al., 2016), has enabled effective resolutions of tens of nanometers (Klimas et al., 2023; Liu et al., 2021), or even in the nanometer range, allowing resolution of single protein structures *in vitro* (Shaib et al., 2023 preprint) (Fig. 4A,B). However, there is one problem that these approaches have in common with ‘classical’ super-resolution microscopy. As the achieved resolution approaches the size range of the biomolecules, it becomes important to deliver the fluorescent label as close as possible to the target. The delivery of dyes at too great a distance from the target, as for example by antibody sandwich labeling, can lead to a clearly measurable ‘labeling error’, a fact that has been recognized in optical super-resolution microscopy (Ries et al., 2012). This also holds true for ExM, and consequently, probes that combine direct labeling by enzymes, such as the SNAP- or Halo-tags, with organic dyes that are linked into the gel have been generated (Thielhorn et al., 2023; Wen et al., 2020). However, labeling errors can also be reduced simply by performing post-expansion staining. If, for example, sandwich antibody staining is performed after expansion instead of before, the label size is effectively ‘shrunk’ by the expansion factor (Chozinski et al., 2016; Gambarotto et al., 2019; Ku et al., 2016; Zwettler et al., 2020). The potential for molecular resolution in fluorescence microscopy by ExM is enormous if the expansion factor, imaging technique and labeling are optimized. However, all this requires tremendous sample preparation in order to achieve an excellent structure preservation, as seen in the example of the centriole (Fig. 4C,D) (Gambarotto et al., 2019).

## Conclusions

Like many new breakthrough techniques, ExM presents a unique set of new challenges that must be overcome to realize its full potential. Foremost, these include the sheer size of the resulting gels. ExM allows the processing of entire animals, such as zebrafish larvae, and thus might in the future provide an alternative to clearing techniques that fix samples and remove scattering tissue components without leading to an expansion of the sample, such as DISCO (Mai et al., 2023) (Fig. 3D), and might become the best approach to image entire animals at highest quality. Of course, expanding the cleared tissues would result in higher resolution, but the ExM gels would be of enormous size, posing challenges to imaging hardware. Furthermore, the imaging of such large samples demands specialized microscopy equipment and software that allow for fast acquisition of the many large, tiled stacks of images, subsequent combination of image stacks by stitching and downstream automated analysis (Chen et al., 2020). Conveniently, this demand overlaps with ongoing efforts in the light-sheet microscopy field, where it has long been realized that large samples require novel solutions for tiling assembly, stitching of two- and three-dimensional images, new objectives and detectors, which allow the imaging of very large fields of view at optimal resolution, as well as the development of software that automatically processes large datasets (Chakraborty et al., 2019; Chen et al., 2020; Preibisch et al., 2009; Voigt et al., 2019).

A longstanding goal that might be realized through ExM is the development of super-resolution methods that are compatible with automated high-throughput microscopy. The development of a protocol that allows for ExM sample preparation to be compatible with 96-well or 384-well plates would be highly desirable, as it would enable the mechanization of the process and avoid sample handling. The first steps in this direction have been made by applying small drops of hydrogel solution to the center of wells in a 96-well plate via a plastic harness (Day et al., 2023 preprint), but further automation would be required. A possible path towards automation might come from photo-induced polymerization (Blatchley et al., 2022; Günay et al., 2023), which could allow the generation of small hydrogels within a larger sample in a directed and controlled manner, or could be combined with microfluidics to improve high-throughput ExM. A further exciting avenue of research is the combination of ExM with emerging spatial *in situ* transcriptomic profiling approaches in intact tissues, as described above (Alon et al., 2021).

A concern that has been raised since the introduction of ExM is the question of whether expansion is isotropic at the nanoscopic level. This has been addressed in a variety of systems with many controls, and isotropic resolution indeed appears to be achievable, even at the level of macromolecular complexes, such as the well-studied centriole (Gambarotto et al., 2019), and single proteins, such as antibodies (Shaib et al., 2023 preprint) (Fig. 4). The structure preservation for proteinaceous complexes and even individual proteins in ExM has thus proven to be excellent. However, it remains unclear how other types of molecule, such as nucleic acids and lipids, withstand the expansion process. Heat- and detergent-based homogenization methods effectively remove all lipids that are not chemically fixed within the specimen, and of course once lipids connected to the gel matrix are pulled apart in the expansion process, the continuity of any membrane will be lost. Similarly, DNA is not digested by the treatments most commonly used in ExM, and artifacts may arise when the proteins decorating DNA and the DNA itself experience expansion differently. At the scale of micrometers, large DNA-containing structures seem to expand faithfully, as spindles appear to be structurally conserved in ExM (Chozinski et al., 2016) and perturbation in interphase nuclei seems to be low (Pownall et al., 2023). In the future, it will be important to determine whether and to what extent subnuclear organization of DNA and DNA-binding proteins is faithfully reproduced in the expanded state at the nanoscale.

In this regard, a thorough analysis of nanoscale perturbations and the development of novel nanoscopic ‘rulers’ that indicate the local expansion factor at the subcellular level in the nanoscopic range serving as *in situ* expansion controls would be highly valuable. Attempts in this direction have been made using DNA origami (Lee et al., 2021; Scheible and Tinnefeld, 2018 preprint) and substrate patterning (Damstra et al., 2023), but nanorulers that could be applied to the sample to detect expansion factors in the local environment would be especially useful.

In summary, ExM is an easily accessible technology that, if properly performed and controlled, allows the resolution of structures smaller than the diffraction limit of light in virtually any biological sample. In the few years since its invention, ExM has already shown remarkable compatibility with super-resolution microscopy approaches and various ‘omics’ techniques, and has been tested in a wide variety of species and even samples from patients. ExM protocols that allow the processing of formalin-fixed, paraffin-embedded tissue samples have been essential in allowing this progress (Bai et al., 2023; Mao et al., 2020; Unnersjö-Jess et al., 2022; Zhao et al., 2017; Cheng et al., 2023). If such sample preparation protocols can be implemented in high-throughput automation and imaging, ExM has the potential to also have a high impact on diagnostics. Furthermore, the fluorescence microscopy field is gearing up to benefit from the potential of ExM. Indeed, ExM images are already included as standards for benchmarking of segmentation algorithms (Cahoon et al., 2017; Halpern et al., 2017), and new fluorescent proteins are being developed specifically with their ExM performance in mind (Campbell et al., 2020, 2022). ExM is here to stay: try it and enjoy the ride.

## Supplementary Material

10.1242/joces.260765_sup1 Supplementary information
