# Supplementary material for: Expanding boundaries – a cell biologist's guide to expansion microscopy
Source: J Cell Sci. 2024 Apr 17;137(7):jcs260765. doi: 10.1242/jcs.260765 (PMC11058692; doi:10.1242/jcs.260765)
Supplement: Supplementary information [file joces-137-260765-s1.pdf]

Table S1. Common expansion microscopy protocols.

Name	Monomer mix	Polymerization	Digestion or denaturation	Expansion factor	Anchoring	Advantage	Reference
Original ExM	2.5% AA, 0.15% BIS, 8.6% SA	0.2% APS, 0.2% TEMED (0.01% 4-HT)	Proteinase K	4.5	Trifunctional label that contains a methacryloyl group	Super-resolution microscopy on conventional microscopes; invention of ExM.	Chen et al., 2015
ExR (expansion revealing)	2.5% AA, 0.075% BIS, 8.6% SA<br><br>13.75% AA, 0.038% BIS<br><br>2.5% AA, 0.038% BIS, 8.6% SA	0.2% APS, 0.2% TEMED, 0.01% 4-HT<br><br>0.025% APS, 0.025% TEMED<br><br>0.025% APS, 0.025% TEMED	SDS	15–20	PFA and AA	Decrowding results in better accessibility of epitopes for antibody staining.	Sarkar et al., 2022
ExT (expansion tomography)	10% AA, 15% AMPS-Na, 0.1% MBA	0.05% APS, 0.01% TEMED	Proteinase K	4	MA–NHS	Polyacrylamide gel with high mechanical stability that allows for sectioning.	Chen et al., 2021b
iExM (iterative ExM)	2.5% AA, 0.2% DHEBA/ 0.2% BAC, 8.6% SA	0.2% APS, 0.2% TEMED	Proteinase K	16–22	Acrydite	Resolution of ~25 nm on conventional microscopes.	Chang et al., 2017
iU-ExM (iterative U-ExM)	Same as U-ExM (see below)	0.1% APS, 0.1% TEMED	SDS/heat	Up to 16	FA and AA	Excellent preservation and high expansion factor.	Louvel et al., 2023
Magnify	10% AA, 34 % SA, 4% DMAA, 0.01% BIS	0.2% APS, 0.2% TEMED, 0.001% 4-HT	SDS/heat	8–10	0.1–0.25% methacrolein	Excellent preservation of antigens. No separate anchoring step.	Klimas et al., 2023
MAP (magnified analysis of the proteome)	20% AA, 0.1% BIS, 7% SA	0.5% APS, 0.5% TEMED	SDS	4	PFA and AA	Expansion of entire organs. Intra- and inter-protein crosslinking is prevented by high AA concentration.	Ku et al., 2016
NIFS (ninefold swelling)	2.5% AA, 8.6% SA, 0.06–0.20% EBIS	0.2% APS, 0.2% TEMED	Proteinase K	9	MA–NHS	Novel superabsorbent hydrogel	Li et al., 2022a
ONE (one-nanometer expansion)	Same as X10 (see below)	Same as X10 (see below)	–	10	–	Maximal resolution	Shaib et al., 2023 preprint
Pan-ExM	19% SA, 10% AA, 0.1% DHEBA	0.25% APS, 0.25% TEMED	SDS/heat	Up to 20	GA	Staining of all proteins	M'Saad and Bewersdorf, 2020
PhotoExM (photoinitiated polymerization of hydrogels for ExM)	3% AA, 16% SA, 0.875% PEGdiAcM, 6% 8-arm, 10 kDa PEG-SH, 0.2% LAP	–	Proteinase K	6.7	AcX	Gels with higher elastic modulus. Amount of expansion is tunable by exposure to light.	Günay et al., 2023

Journal of Cell Science • Supplementary information

ProExM (protein retention ExM)	2.5% AA, 0.15% BIS, 8.6% SA	0.15% APS, 0.15% TEMED (0.01% 4-HT)	Proteinase K, LysC	4	AcX, MA–NHS, glutaraldehyde	Proteins are anchored. Allows use of conventional antibodies.	Tillberg et al., 2016
Tetra-gel ExM	Mix of monomer 1 and 2 in a 1:1 ratio	–	Proteinase K	3.0–3.5	NHS–azide	Non-radical-based and results in a more homogeneous network structure.	Gao et al., 2021
T-Rev ExM (thermoresponsive reversible ExM)	LCST hydrogels: 8.0% AA, 8.4% N-isopropyl AA, 8.9% N,N-diethylacrylamide, 2.6% N-tert-butylacrylamide, 0.01% BIS<br><br>UCST hydrogels: 45% sulfobetaine, 0.07% BIS	0.5% APS, 0.5% TEMED	Proteinase K	Adjustable up to 3.7	NAS	Allows thermal adjustment of the expansion factor.	Kang et al., 2021
TREx (Tenfold robust expansion microscopy)	1.1 M SA, 2.0 M AA	1.5 mg/ml APS, 1.5 mg/ ml TEMED	Proteinase K	~10	AcX	Increased resolution of 25–30 nm	Damstra et al., 2022
U-ExM (ultrastructure ExM)	10% AA, 0.1% BIS, 19% SA	0.5% APS, 0.5% TEMED	SDS	4	FA and AA	Visualization of preserved ultrastructures	Gambarotto et al., 2019
uniExM (unified ExM)	As in original ExM or TREx	As in original ExM or TREx	–	4–7	glycidyl methacrylate	Low cost and fixes multiple molecular species.	Cui et al., 2023
X10	26.7% DMAA, 6.4% SA	0.36% KPS, 0.4% TEMED	Proteinase K	10	AcX	Increased resolution of 25–30 nm	Truckenbrodt et al., 2018
X10ht (X10 heat-treated)	Same as X10	Same as X10	Autoclave 110°C 5% Triton X-100, 1% SDS, pH 8.0	10	AcX	Thorough homogenization results in higher fluorescence intensity.	Saal et al., 2023
ZOOM (Zoom by hydrogel conversion microscopy)	30% AA, 0.01% BIS	0.5% APS, 0.5% TEMED	SDS and boric acid	8	NAS	Adjustable expansion factor	Park et al., 2019

AA, acrylamide; AMPS-Na, sodium 2-acryla-mido-2-methyl-1-propanesulfonic acid; APS, ammonium persulfate; BAC, *N,N′*-cystaminebisacrylamide; BIS, *N,N′*-methylenebis(acrylamide); DHEBA, *N,N′*-(1,2-dihydroxyethylene)bisacrylamide; DMAA, *N,N*-dimethylacrylamide; EBIS, *N,N*-ethylenebis(acrylamide); FA, formaldehyde; GA, glutaraldehyde; KPS, potassium persulfate; LAP, lithium phenyl-2,4,6-trimethylbenzoylphosphinate; LCST, lower critical solution temperature; MA-NHS, methacrylic acid *N*-hydroxysuccinimide ester; MBA, *N,N'*-methylenebis(acrylamide); NAS, acrylic acid *N*-hydroxysuccinimide ester; PEGdiAcM, polyethylene glycol–diacrylamide crosslinker; PEG-SH, polyethylene glycol with thiol-groups; PFA, paraformaldehyde; SA, sodium acrylate; TEMED, *N,N,N′,N′*-tetramethylethylenediamine; SDS, sodium dodecyl sulfate; UCST, upper critical solution temperature.

Table S2. Useful quantitative studies of parameters in ExM.

Type of information	Reference	Location in article
Homogenization versus number of proteins identified in mass spectrometry	Drelich et al., 2021	
Gel composition versus expansion factor	Damstra et al., 2022	Figure 1
Salt content versus expansion factor	Damstra et al., 2022; Park et al., 2023	Figure 4, supplement 1A; Figure S2
Crosslinker concentration versus expansion factor	Chen et al., 2015; Damstra et al., 2022; Hu et al., 2020; Li et al., 2022a	Figure S5; Figure 1; Figure 2; Figure 2
Light transmission of gels	Chen et al., 2015	Figure S2
Fluorophore retention	Chen et al., 2015; Tillberg et al., 2016	Table S1; Figure 1
Digestion time	Chozinski et al., 2016a	Figures S13, S14 and S15
Proteases, expansion and signal retention	Yu et al., 2020	Figure 11C
Surface grafting and gel linkage	Chozinski et al., 2016	Figures S1, S10, S11, S16, S17 and S20
Mechanical properties: elastic modulus	Chen et al., 2021b	Figure 2
Mechanical properties: stiffness	Park et al., 2019	Figure S5
Factors determining expansion of different bacterial species	Lim et al., 2019	Figure S1
Prevention of gel shrinkage in ionic buffers	Fan et al., 2021	Figures 3 and 4

Journal of Cell Science • Supplementary information

Table S3. Staining methods.

Method	Targeted biomolecules	Target specificity	Polymer anchoring	Detection	Specialities	Limitations	Reference
BODIPY TR ceramide	Sphingolipid incorporates into membranes. Originally targeted nuclear envelope but can target cellular membranes.	Incorporation of ceramide in membranes.	Similar to that in U-ExM	BODIPY TR ceramide	Rather general membrane staining that can be applied to gels without specific priming.	Staining done after expansion. Might not work in harshly permeabilized or homogenized membranes.	Liffner and Absalon, 2021
Click-ExM	Glycans, lipids, proteins, DNA and small molecules containing an azide (such as azide-afatinib).	Metabolic or genetic labeling (alkyne or azide). Click reaction with functionalized biotin followed by Streptavidin staining of biotin.	Primary amines on Streptavidin linked via AcX or GA	Streptavidin–fluorescent dye conjugate	Signal amplification by iterative biotin-trimer–Streptavidin binding reactions. Compatible with immunosignal hybridization chain reaction (isHCR) on Streptavidin. Other pairings are possible (e.g. azide–FLAG and anti-FLAG antibody).	Not more than two different click targets are possible, but the method is compatible with antibody-based staining.	Sun et al., 2021
ChromExM	Chromatin/DNA	Metabolic labeling with f-*ara*-EdU [(2′S)-2′-deoxy-2′-fluoro-5-ethynyluridine]. Click reaction with picolyl azide.	FA/AA	AZ555 picolyl azide	Bright photostable nuclear staining.	Metabolic labeling requires injection of embryos with f-*ara*-EdU.	Pownall et al., 2023
Ex FISH	RNA and DNA	Specific RNA FISH probes complementary to retained cellular RNA	LabelX: Label-IT nucleotide reactive label conjugated to AcX	Fluorophore conjugated to FISH probe	ExFISH staining done after expansion. Allows decrowding. Compatible with DNA hybridization chain reaction amplifier technique for tissue imaging. Serial ExFISH with different probes is possible. Compatible with proExM.	FISH probes have to be specifically synthesized. Gels have to be re-embedded in neutral polyacrylamide gel for serial ExFISH. Lower expansion factor (3×) due to salt contained in hybridization buffer.	Chen et al., 2016

Journal of Cell Science • Supplementary information

Method	Targeted biomolecules	Target specificity	Polymer anchoring	Detection	Specialities	Limitations	Reference
Expansion-SABER	Specific proteins stained by antibodies	Primary antibody modified with a single-stranded DNA	Complementary concatemer strand modified with acrydite moiety	Concatemer hybridizes with DNA on the antibody; it contains nucleotide sequence repeats for amplified signals, which are bound by fluorescent imager oligonucleotides.	Signal amplification due to the multiple imager binding sites on one concatemer. Multiplexing possible through dehybridization of DNA. Post-expansion staining can be used.	Synthesis of concatemers needed. Antibodies need to be linked to specific DNA strands. Expansion factor only 3×, due to re-embedding of gel in uncharged second gel.	Saka et al., 2019
FLARE	Glycans or proteins (with NHS-ester, similar to panExM)	Chemical modification of carbohydrates, introduction of aldehydes that allow coupling to functionalized fluorophores.	FA/AA functionalization of primary amines prior to staining	Hydrazide-functionalized fluorophores (for glycans) or NHS-ester (for proteins)	Non-metabolic labeling of glycans. Compatible with immunolabeling and FISH. Staining done after gelation.	Carbohydrates need to be anchored to gel, which is only possible for glycans. Functionalization for polymerization uses the same amines as NHS-ester staining. Fixed order of staining for carbohydrates, amines and antibodies	Mao et al., 2020
Fluorescent protein expression	Target proteins	Genetic labeling with fluorescent protein tag	Similar to protein retention method. AcX or GA modification	Fluorescence of fluorescent protein	No extra labeling step required for fluorescent protein-modified cell lines. Compatible with PALM.	Not compatible with denaturing ExM, such as MAP or U-ExM.	Chozinski et al., 2016; Tillberg et al., 2016
LExM	Phosphatidyl-choline and other choline-containing lipids	Metabolic alkynyl labeling with propargylcholine. Trifunctional LexM reagent [fluorophore, anchor and reactive azides for azide–alkyne cycloaddition (CuAAC)].	Methacrylamide part of the LExM reagent	Fluorophore part of the LExM reagent	Direct grafting due to methacrylamide group. Fluorophores specifically suited for lipid imaging.	Metabolic labeling and click reaction needed.	White et al., 2022

Journal of Cell Science • Supplementary information

Method	Targeted biomolecules	Target specificity	Polymer anchoring	Detection	Specialities	Limitations	Reference
LR-ExM	Specific protein	Genetic labeling with CLIP-tag or SNAP-tag.<br>Trifunctional linker contains benzylguanine or benzylcytosine for SNAP/CLIP-tagging and biotin or digoxigenin for detection.	Methacrylamide group contained in trifunctional linker	Staining with Streptavidin–dye conjugate after expansion	Fluorescence is greatly retained, as the reporter is directly linked to the gel and the dye introduced after expansion.<br>Two-color LR-ExM possible by mixing reporters (biotin and digoxigenin).<br>Exchanging the BG/BC for NHS, the linker can be used for antibody labeling.	Only the target structures are retained in the gel.<br>Synthesis of trifunctional linker needed.	Shi et al., 2021
mCLING	Lipid membranes	mCLING is incorporated into membrane due to a palmitoyl tail	AcX or GA fixation allows crosslinking of primary amines contained in seven lysines of mCLING	Fluorophore covalently linked to mCLING molecule	Commercial membrane-staining molecule that contains all the features needed for ExM.<br>Works for live cells (plasma membrane) and fixed cells (plasma membrane and organelle membranes).	General limitations of lipid membrane fixation	Damstra et al., 2022; Götz et al., 2020b
mExM with pGk5b	Lipid membranes	Conjugated lipid tail of probe intercalates into lipid membrane (e.g. palmitoyl).	Primary amines on the lysine chain that is part of the probe can be linked, for example, with AcX.	Probe contains a biotin handle, which is detected by Streptavidin–dye conjugate (post gelation).	Signal amplification possible by re-incubation with fluorescent biotin conjugates.<br>Compatible with post-expansion antibody staining for protein targets.<br>Use of (D)-lysine prevents probe from enzymatic digestion.	Probe is lost during permeabilization for conventional immunofluorescence.<br>Only compatible with post-expansion staining (similar to U-ExM or MAP).	Karagiannis et al., 2019 preprint
Original ExM	Specific proteins stained by antibody	Oligonucleotide strand on the trifunctional linker hybridizes to complementary sequence attached to an affinity tag (e.g. an antibody).	Methacryloyl group on the trifunctional linker	Fluorophore on the trifunctional linker	Trifunctional linker contains all the necessary functions.<br>Theoretically compatible with other biomolecules if they can be coupled to oligonucleotides.	Synthesis of specific trifunctional linker and complementary single-stranded oligonucleotide required.	Chen et al., 2015

Journal of Cell Science • Supplementary information

Method	Targeted biomolecules	Target specificity	Polymer anchoring	Detection	Specialities	Limitations	Reference
Pan-ExM	All proteins (primary amines), palmitoylated proteins (functionalized by azide group), or cysteine-rich proteins	Dye–NHS-ester can label all primary amines after expansion. Metabolic labeling of palmitoylated proteins with palmitic acid azide, click reaction with alkyne–dye conjugate. Maleimide reacts with cysteines in cysteine-rich proteins.	Proteins retained by FA/AA anchoring	NHS-ester–dye, alkyne–dye or maleimide–dye conjugates incubated after expansion	Compatible with immunostaining. Post-expansion labeling, reduced linkage error and decrowded protein-dense areas.	Functionalization for polymerization uses the same amines as NHS-ester staining.	M'Saad and Bewersdorf, 2020
p-ExM	Unspecific cellular structures (in neurons)	Plasmonic-fluor nanolabel	BSA–biotin layer on nanoparticle is anchored with AcX	IR-650 fluorescent dye on the nanoparticle	Very high signal retention compared to fluorophore alone	Binding of particles to hippocampal neurons reported. Method might not work in other cell types.	Rathi et al., 2023
Plasmonic nanoparticles	Specific antigens	Antibody-based labeling. Linking of plasmonic nanoparticle to secondary antibody via biotin–Streptavidin	Similar to proExM	Near infrared Raman dye on the particle	Can be transferred to any antibody-based labeling	Detection based on surface-enhanced Raman scattering spectroscopy (SERS) and dark-field spectroscopy.	Artur et al., 2018
Poly(dT)	mRNA with polyadenylation	15-nucleotide poly(dT) oligomer hybridizes to poly(A) tail	Acrydite moiety on the poly(dT) probe	Detection of RNA as in classic FISH (additional, specific probe with fluorophore needed).	Compatible with MERFISH	Original publication used technique for clearing not expansion	Moffitt et al., 2016
ProExM	Specific proteins	Antibody detects antigen before expansion	AcX, MA-NHS or GA modifies amines on proteins (i.e., antibodies).	Fluorophore introduced by classical immuno-labeling	Fluorescent proteins survive enzymatic digestion.<br>No need for special synthesized components.	Incompatible with rhodamine dyes (which do not survive radical polymerization).	Chozinski et al., 2016; Tillberg et al., 2016
smiFISH with ExM	Specific RNAs	RNA duplex (primary and secondary probe) containing a single-strand overhang complementary to the target RNA	Secondary probe contains acrydite modification	Secondary probe is conjugated to fluorophore.	Detection and staining are done before expansion.		Tsanov et al., 2016
Sphingolipid ExM	Sphingolipid (modified with amino group)-containing membranes	Metabolic labeling with amino-azido-modified ceramide. Click reaction of DBCO–dye conjugate with amino-ceramide.	GA fixation allows crosslinking of amino-ceramide	DBCO–dye conjugate reacts with amino-azido-ceramide	Compatible with antibody staining (proExM)	Synthesis of modified ceramide probe required.	Götz et al., 2020b

Journal of Cell Science • Supplementary information

Method	Targeted biomolecules	Target specificity	Polymer anchoring	Detection	Specialities	Limitations	Reference
TRITON	Actin, phospholipid bilayers, specific proteins or nucleic acids	Phalloidin–TRITON for actin. DSPE–TRITON for phospholipid bilayers. Antibody–TRITON for proteins. DNA–TRITON for nucleic acids.<br>Specific TRITON probe contains a reactive handle to conjugate to different molecules (small molecules and antibodies, DNA oligonucleotides).	Acrylamide group in the TRITON probe	Fluorescent dye part of the TRITON probe	Instead of a fluorescent dye, oligonucleotide barcodes can be used, allowing post-expansion labeling with complementary oligonucleotide–dye conjugate, which prevents signal loss during polymerization.<br>Variation with post-digestion introduction of fluorophore via click chemistry.	Re-embedding in neutral hydrogel needed, which leads to smaller expansion factor of 3×.<br>TRITON probe needs to be synthesized and linked to reporter.	Wen et al., 2020; Wen et al., 2021; Wen et al., 2023b
U-ExM/MAP	Proteins<br>(all molecules with a primary amine)	Antibody detects antigen post expansion	Molecules that contain primary amines are functionalized using formaldehyde with acrylamide or AcX	Fluorophore introduction via secondary antibody	Proteins are retained and denatured. Post-expansion staining allows multicolor and repetitive staining.<br>Decrowding.<br>Reduction in linkage error.	Only structures with primary amines are linked to the gel.<br>Fluorescent proteins are denatured and need to be visualized with antibodies.<br>Requires an antibody that detects denatured proteins (such as in western blotting).	Gambarotto et al., 2019; Ku et al., 2016; Tillberg et al., 2016
Unclearing Microscopy	Pan-staining	Chromogen deposition<br>(DAB or metallic silver)	Similar to pan-ExM	Fluorescence detection not needed; chromogen is visible with the naked eye, phone camera or phase-contrast microscopy.	High educational value: seeing cells and tissue without a lens or microscope.		M'Saad et al., 2022 preprint

BC, benzylcytosine; BG, benzylguanine; BODIPY, boron dipyrromethene; BODIPY TR, (N-((4-(4,4-difluoro-5-(2-thienyl)-4-bora-3a,4a-diaza-s-indacene-3-yl)phenoxy)acetyl)sphingosine); BSA, bovine serum albumin; DAB, 3,3′-diaminobenzidine; DBCO, 6-oxo-6-(dibenzo[b,f]azacyclooct-4-yn-1-yl)-caproic acid, a chemical group that allows copper-free click chemistry; DSPE, 1,2-distearoyl-sn-glycero-3-phosphoethanolamine; MERFISH, multiplexed error-robust fluorescence *in situ* hybridization; p-ExM, plasmon-enhanced ExM; pGk5b, palmitoyl-G-KKKKK-biotin.

Journal of Cell Science • Supplementary information

Table S4. Organisms used with ExM.

Category	Sample species and tissue	Reference
**Model Systems**		
Yeast	*Saccharomyces cerevisiae*	Chen et al., 2021a; Hinterndorfer et al., 2022
Worm	*Caenorhabditis elegans*	Yu et al., 2020; Yu et al., 2022
Fly	*Drosophila* central nervous system tissue	Mosca et al., 2017
	Drosophila embryos, larval brains, larval and adult body walls	Jiang et al., 2018
	Drosophila wing	Steib et al., 2022
	Drosophila synaptonemal complex	Cahoon et al., 2017
Fish	*Danio rerio*	Freifeld et al., 2017
	D. rerio embryo	Steib et al., 2022
	D. rerio larvae	Sim et al., 2022 preprint; Wang and Wang, 2023
	D. rerio, larval and embryonic	Freifeld et al., 2017
Mouse	murine organoids	Blatchley et al., 2022
	Mus musculus embryo	Sim et al., 2022 preprint; Steib et al., 2022
Human	human airway cells infection model	Nijenhuis et al., 2021 preprint
	human erythrocytes	Hou et al., 2023
	human organoids	Blatchley et al., 2022; Rodriguez-Gatica et al., 2022
**Microorganisms**		
Algae	*Chlamydomonas*	Gambarotto et al., 2019
Bacteria	*Acetobacter tropicalis*	Lim et al., 2019
	Acidaminococcus intestini	Lim et al., 2019
	Bacillus subtilis	Middelhauve et al., 2023
	Bacteroides finegoldii	Lim et al., 2019
	Bacteroides ovatus	Lim et al., 2019
	Bifidobacterium breve	Lim et al., 2019
	Chlamydia trachomatis	Götz et al., 2020b; Kunz et al., 2019
	Citrobacter sp.	Lim et al., 2019
	Clostridium innocuum	Lim et al., 2019
	Escherichia coli	Lim et al., 2019; Cheng et al., 2023
	Lactobacillus plantarum	Lim et al., 2019
	Neisseria gonorrhoeae	Götz et al., 2020b
	Parabacteroides distasonis	Lim et al., 2019
	Pseudomonas aeruginosa	Cheng et al., 2023
	Salmonella enterica	Lim et al., 2019
	Simkania negevensis	Götz et al., 2020b

Journal of Cell Science • Supplementary information

**Category**	**Sample species and tissue**	**Reference**
	Staphylococcus aureus	Kunz et al., 2021; Cheng et al., 2023
	Staphylococcus epidermidis	Cheng et al., 2023
	Streptococcus pneumoniae	Cheng et al., 2023
**Other organisms**		
Birds	*Gallus gallus domesticus,* chick embryo	Wilmerding et al., 2023
	Taeniopygia (Zebra finches)	Düring et al., 2019
Fish	*Oryzias melastigma* (Medaka)	Wang and Wang, 2023
Fungi	*Aspergillus fumigatus*	Götz et al., 2020a
	Candida albicans	Cheng et al., 2023
	Fusarium oxysporum	Götz et al., 2020a
	Ustilago maydis	Götz et al., 2020a
	Schizosaccharomyces pombe	Hinterndorfer et al., 2022
Insects	*Apis mellifera* (Honey bee)	Kraft et al., 2023
Parasites	*Giardia lamblia*	Halpern et al., 2017
	Leishmania major	Gorilak et al., 2021
	Plasmodium falciparum	Liffner and Absalon, 2021; Liffner et al., 2023; Rashpa and Brochet, 2022
	Toxoplasma gondii	Dave et al., 2022; Engelberg et al., 2022; Gambarotto et al., 2021; Sparvoli et al., 2022; Tosetti et al., 2020
	Trypanosoma brucei	Gambarotto et al., 2019; Gorilak et al., 2021; Kalichava and Ochsenreiter, 2021
	Trypanosoma cruzi	Campbell and de Graffenried, 2023
Planarians	*Schmidtea mediterranea*	Lim et al., 2019
Plants	*Arabidopsis* seedlings	Hawkins et al., 2023
	Arabidopsis thaliana zygotes	Kao and Nodine, 2019; Kao and Nodine, 2021
	Spinacia oleracea chloroplasts	Bos et al., 2024
	Tobacco suspension culture cells (BY2, from *Nicotiana tabacum cv.*)	Hawkins et al., 2023
Viruses	Bacteriophage T4	Gambarotto et al., 2021

Supplementary References

Artur, C. G., Womack, T., Zhao, F., Eriksen, J. L., Mayerich, D. and Shih, W.-C. (2018). Plasmonic nanoparticle-based expansion microscopy with surface-enhanced Raman and dark-field spectroscopic imaging. *Biomed. Opt. Express* **9**, 603-615.

Bos, P. R., Berentsen, J. and Wientjes, E. (2024). Expansion microscopy resolves the thylakoid structure of spinach. *Plant Physiol.*, **194**, 347-358.

Campbell, P. C. and de Graffenried, C. L. (2023). Morphogenesis in Trypanosoma cruzi epimastigotes proceeds via a highly asymmetric cell division. *PLOS Negl. Trop. Dis.* **17**, e0011731.

Chen, L., Yao, L., Zhang, L., Fei, Y., Mi, L. and Ma, J. (2021a). Applications of Super Resolution Expansion Microscopy in Yeast. *Front. Phys.* **9**, 650353.

Chen, R., Chen, R., Cheng, X., Cheng, X., Zhang, Y., Zhang, Y., Yang, X., Yang, X., Wang, Y., Wang, Y., et al. (2021b). Expansion tomography for large volume tissue imaging with nanoscale resolution. *Biomed. Opt. Express* **12**, 5614-5628.

Dave, N., LaFavers, K. and Arrizabalaga, G. (2022). The Dually Localized EF-Hand Domain-Containing Protein TgEFP1 Regulates the Lytic Cycle of Toxoplasma gondii. *Cells* **11**, 1709.

Engelberg, K., Bechtel, T., Michaud, C., Weerapana, E. and Gubbels, M.-J. (2022). Proteomic characterization of the Toxoplasma gondii cytokinesis machinery portrays an expanded hierarchy of its assembly and function. *Nat. Commun.* **13**, 4644.

Fan, Y., Lim, Y., Wyss, L. S., Park, S., Xu, C., Fu, H., Fei, J., Hong, Y. and Wang, B. (2021). Mechanical expansion microscopy. In *Methods in Cell Biology*, pp. 125-146. Elsevier.

Freifeld, L., Odstrcil, I., Förster, D., Ramirez, A., Gagnon, J. A., Randlett, O., Costa, E. K., Asano, S., Celiker, O. T., Gao, R., et al. (2017). Expansion microscopy of zebrafish for neuroscience and developmental biology studies. *Proc. Natl. Acad. Sci. USA* **114**, E10799-E10808.

Gambarotto, D., Hamel, V. and Guichard, P. (2021). Ultrastructure expansion microscopy (U-ExM). In *Methods in Cell Biology*, pp. 57-81. Elsevier.

Gorilak, P., Pružincová, M., Vachova, H., Olšinová, M., Schmidt Cernohorska, M. and Varga, V. (2021). Expansion microscopy facilitates quantitative super-resolution studies of cytoskeletal structures in kinetoplastid parasites. *Open Biol.* **11**, 210131.

Götz, R., Panzer, S., Trinks, N., Eilts, J., Wagener, J., Turrà, D., Di Pietro, A., Sauer, M. and Terpitz, U. (2020a). Expansion Microscopy for Cell Biology Analysis in Fungi. *Front. Microbiol.* **11**.

Götz, R., Kunz, T. C., Fink, J., Solger, F., Schlegel, J., Seibel, J., Kozjak-Pavlovic, V., Rudel, T. and Sauer, M. (2020b). Nanoscale imaging of bacterial infections by sphingolipid expansion microscopy. *Nat. Commun.* **11**, 6173.

Journal of Cell Science • Supplementary information

Hawkins, T. J., Robson, J. L., Cole, B. and Bush, S. J. (2023). Expansion Microscopy of Plant Cells (PlantExM). In *The Plant Cytoskeleton: Methods and Protocols* (ed. Hussey, P. J.) and Wang, P.), pp. 127-142. New York, NY: Springer US.

Hinterndorfer, K., Laporte, M. H., Mikus, F., Tafur, L., Bourgoint, C., Prouteau, M., Dey, G., Loewith, R., Guichard, P. and Hamel, V. (2022). Ultrastructure expansion microscopy reveals the cellular architecture of budding and fission yeast. *J. Cell Sci.* **135**, jcs260240.

Hou, M., Xing, F., Yang, J., Hu, F., Pan, L. and Xu, J. (2023). Molecular Resolution Mapping of Erythrocyte Cytoskeleton by Ultrastructure Expansion Single-Molecule Localization Microscopy. *Small Methods* **7**, 2201243.

Hu, Y., Chu, X., Chen, T., Pan, Q., Liu, C., Yi, J. and Chu, X. (2020). Improving resolving ability of expansion microscopy by varying crosslinker concentration. *Chem. Commun.* **56**, 4176-4179.

Kalichava, A. and Ochsenreiter, T. (2021). Ultrastructure expansion microscopy in Trypanosoma brucei. *Open Biol.* **11**, 210132.

Kang, S., Park, S., Song, H., Choi, D., Park, H.-E., Ahn, B. H., Kim, S.-Y. and Lee, Y. (2021). Expansion Microscopy with a Thermally Adjustable Expansion Factor Using Thermoresponsive Biospecimen–Hydrogel Hybrids. *ACS Appl. Mater. Interfaces* **13**, 28962-28974.

Kao, P. and Nodine, M. D. (2021). Application of expansion microscopy on developing Arabidopsis seeds. In *Methods in Cell Biology*, pp. 181-195. Elsevier.

Kraft, N., Muenz, T. S., Reinhard, S., Werner, C., Sauer, M., Groh, C. and Rössler, W. (2023). Expansion microscopy in honeybee brains for high-resolution neuroanatomical analyses in social insects. *Cell Tissue Res*. **393**, 489-506.

Kunz, T. C., Götz, R., Sauer, M. and Rudel, T. (2019). Detection of Chlamydia Developmental Forms and Secreted Effectors by Expansion Microscopy. *Front. Cell. Infect. Microbiol.* **9**, 276.

Kunz, T. C., Rühling, M., Moldovan, A., Paprotka, K., Kozjak-Pavlovic, V., Rudel, T. and Fraunholz, M. (2021). The Expandables: Cracking the Staphylococcal Cell Wall for Expansion Microscopy. *Front. Cell. Infect. Microbiol.* **11**, 644750.

Lim, Y., Shiver, A. L., Khariton, M., Lane, K. M., Ng, K. M., Bray, S. R., Qin, J., Huang, K. C. and Wang, B. (2019). Mechanically resolved imaging of bacteria using expansion microscopy. *PLOS Biol.* **17**, e3000268.

Middelhauve, V., Siebrasse, J. P. and Kubitscheck, U. (2023). Expansion Microscopy of Bacillus subtilis. In *Antibiotics: Methods and Protocols* (ed. Sass, P.), pp. 191–202. New York, NY: Springer US.

Journal of Cell Science • Supplementary information

Moffitt, J. R., Hao, J., Bambah-Mukku, D., Lu, T., Dulac, C. and Zhuang, X. (2016). High-performance multiplexed fluorescence in situ hybridization in culture and tissue with matrix imprinting and clearing. *Proc. Natl. Acad. Sci. USA* **113**, 14456-14461.

Nijenhuis, W., Damstra, H. G. J., Grinsven, E. J. van, Iwanski, M. K., Praest, P., Soltani, Z. E., Grinsven, M. M. P. van, Brunsveld, J. E., Kort, T. de, Rodenburg, L. W., et al. (2021). Optical nanoscopy reveals SARS-CoV-2-induced remodeling of human airway cells. *BioRxiv*, 2021.08.05.455126.

Park, H., Choi, D., Park, J. S., Sim, C., Park, S., Kang, S., Yim, H., Lee, M., Kim, J., Pac, J., et al. (2019). Scalable and Isotropic Expansion of Tissues with Simply Tunable Expansion Ratio. *Adv. Sci.* **6**, 1901673.

Park, H. H., Choi, A. A. and Xu, K. (2023). Size-Dependent Suppression of Molecular Diffusivity in Expandable Hydrogels: A Single-Molecule Study. *J. Phys. Chem. B* **127**, 3333-3339.

Rashpa, R. and Brochet, M. (2022). Expansion microscopy of Plasmodium gametocytes reveals the molecular architecture of a bipartite microtubule organisation centre coordinating mitosis with axoneme assembly. *PLOS Pathog.* **18**, e1010223.

Rathi, P., Gupta, P., Debnath, A., Baldi, H., Wang, Y., Gupta, R., Raman, B. and Singamaneni, S. (2023). Plasmon-Enhanced Expansion Microscopy. *Nano Lett.* **23**, 5654–5662.

Rodriguez-Gatica, J. E., Iefremova, V., Sokhranyaeva, L., Yeung, S. W. C. A., Breitkreuz, Y., Brüstle, O., Schwarz, M. K. and Kubitscheck, U. (2022). Imaging three-dimensional brain organoid architecture from meso- to nanoscale across development. *Development* **149**, dev200439.

Saal, K. A., Shaib, A. H., Mougios, N., Crzan, D., Opazo, F. and Rizzoli, S. O. (2023). Heat denaturation enables multicolor X10-STED microscopy. *Sci. Rep.* **13**, 5366.

Sparvoli, D., Delabre, J., Penarete-Vargas, D. M., Kumar Mageswaran, S., Tsypin, L. M., Heckendorn, J., Theveny, L., Maynadier, M., Mendonça Cova, M., Berry-Sterkers, L., et al. (2022). An apical membrane complex for triggering rhoptry exocytosis and invasion in Toxoplasma. *EMBO J.* **41**, e111158.

Steib, E., Tetley, R., Laine, R. F., Norris, D. P., Mao, Y. and Vermot, J. (2022). TissUExM enables quantitative ultrastructural analysis in whole vertebrate embryos by expansion microscopy. *Cell Rep. Methods* **2**, 100311.

Tosetti, N., Dos Santos Pacheco, N., Bertiaux, E., Maco, B., Bournonville, L., Hamel, V., Guichard, P. and Soldati-Favre, D. (2020). Essential function of the alveolin network in the subpellicular microtubules and conoid assembly in Toxoplasma gondii. *eLife* **9**, e56635.

Wang, M. and Wang, W.-X. (2023). Nanoscale Whole-Body Expansion Microscopy Revealed the Early Skeletal Developmental Malformation Induced by Silver Nanoparticles. *Environ. Sci. Technol. Lett.* **10**, 471-477.

Journal of Cell Science • Supplementary information

Wen, G., Vanheusden, M., Leen, V., Rohand, T., Vandereyken, K., Voet, T. and Hofkens, J. (2021). A Universal Labeling Strategy for Nucleic Acids in Expansion Microscopy. *J. Am. Chem. Soc.* **143**, 13782-13789.

Wilmerding, A., Espana-Bonilla, P., Giakoumakis, N.-N. and Saade, M. (2023). Expansion microscopy of the chick embryo neural tube to overcome molecular crowding at the centrosomes-cilia. *STAR Protoc.* **4**, 101997.

Yu, C.-C. (Jay), Barry, N. C., Wassie, A. T., Sinha, A., Bhattacharya, A., Asano, S., Zhang, C., Chen, F., Hobert, O., Goodman, M. B., et al. (2020). Expansion microscopy of C. elegans. *eLife* **9**, e46249.

Yu, C.-C. (Jay), Orozco Cosio, D. M. and Boyden, E. S. (2022). ExCel: Super-Resolution Imaging of C. elegans with Expansion Microscopy. In *C. elegans: Methods and Applications* (ed. Haspel, G. and Hart, A. C.), pp. 141-203. New York, NY: Springer US.
